# Demographic and structural indicators of temporomandibular joint osteoarthritis in asymptomatic adults: a cone-beam computed tomography-based multivariate analysis

**DOI:** 10.1007/s11282-026-00944-4

**Published:** 2026-06-18

**Authors:** Daniela Lopera-Arango, Luis Gonzaga López-Buriticá, Sergio Iván Tobón-Arroyave, Clara Inés Saldarriaga-Naranjo, Julián González-García, Lina María Franco-González

**Affiliations:** 1 Graduate Oral and Maxillofacial Surgery Program, Faculty of Dentistry, University of Antioquia, Medellín, Colombia; 2 Department of Radiology, RADEX 3D Specialized Radiology Center, CES University, Medellín, Colombia; 3 Department of Oral Radiology, Radiología y Diagnósticos Odontológicos RDO, Pachuca, Mexico; 4 Laboratory of Immunodetection and Bioanalysis, Faculty of Dentistry, University of Antioquia, Calle 70 N° 52-21, Medellín, Colombia

**Keywords:** Cone beam computed tomography, Diagnostic imaging, Osteoarthritis, Risk assessment, Temporomandibular joint

## Abstract

**Objective:**

To investigate demographic, morphologic, and morphometric variables associated with early or subclinical imaging-based osteoarthritic structural changes of temporomandibular joint (TMJ).

**Methods:**

Cone-beam computed tomography scans of 396 TMJs from 198 asymptomatic individuals (75 males, 123 females) were analyzed. TMJs were classified as normal-appearing, indeterminate for osteoarthritis (OA), or affected by OA based on condyle, and fossa/eminence morphology. Univariate and multivariate logistic regression models assessed the association of 5 patient-level and 20 TMJ-level variables with the indeterminate or affected by OA status, adjusting for confounding.

**Results:**

Prevalence rates of the indeterminate and affected by OA statuses were 25.80% and 33.30% at the patient-level and 25.30% and 24.50% at the TMJ-level, respectively. Whereas complete/partial edentulism was the only strong/independent indicator for indeterminate for OA category, strong indicators associated with the affected by OA status included age > 38.50 years, complete/partial edentulism, Class II skeletal relationship, horizontal condylar angle ≥ 24.14 degrees, condylar height ≤ 16.00 mm, sagittal glenoid fossa width > 16.95 mm, sagittal articular eminence angle ≤ 45.78 degrees, glenoid fossa roof thickness > 1.23 mm (coronal) and > 1.20 mm (sagittal).

**Conclusions:**

CBCT imaging provides a reliable framework for identifying both established and early osteoarthritic changes in the TMJ of asymptomatic adults. Indeterminate cases, often marked by flattening or sclerosis, should be regarded as transitional stages that warrant closer monitoring.

## Introduction

Temporomandibular joint (TMJ) is a bilateral joint located between the mandible and the temporal bone [1]. It possesses both bone and soft tissue components, forming a unique structure that works in harmony with the masticatory muscles and teeth [2], so that the direction and magnitude of the movements are controlled by the muscles, ligaments, the shape of the articular surfaces, and the occlusion [3]. The available evidence suggests that TMJ morphology varies among individuals and can exhibit large differences between left and right sides of the same individual [3, 4]. It has been acknowledged that the TMJ retains its remodeling capability even after growth has ceased [5, 6] owing to its outstanding adaptability to functional and mechanical demands [6, 7]. Therefore, in adulthood, bone structures undergo changes, which when they exceed the limit of normal variations, might lead to the occurrence of pathological disturbances [8] known as osteoarthritic bony changes, which include flattening, erosion, presence of osteophytes, deviations in shape, subcortical sclerosis, and/or subchondral cysts [9, 10].

All these morphological and dimensional variations have been evaluated regarding different factors, leading to the conclusion that the forces exerted on the TMJ may be associated with alterations of cortical and subcortical bone components [8, 11], as well as articular cartilage [11]. Notwithstanding, the results obtained from diverse researches have been inconsistent, possibly for reasons attributable to differences in methodological aspects such as ethnicity, case selection, sample size, imaging techniques, variables analyzed, and statistical methods, as well as discrepancies in the diagnostic criteria used in the evaluations, which may hamper the scientific evidence base for the interpretation of the results. Likewise, while the majority of investigations has concluded that certain potential predictors evaluated individually such as sex, age, craniofacial patterns, dental status, and occlusal variations (i.e., discrepancies, forces, and wearing), significantly affect the morphology and dimensions of the articular structures [6, 10–16] leading to osteoarthritic changes [10], there is only scant evidence on those parameters that could affect this relationship as possible confounding factors or on their synergistic interaction effects [11–13], so it is not possible to preclude unequivocal conclusions.

Imaging evaluation of articular structures is of utmost importance for the detection of osteoarthritic changes in the TMJ [10, 16]. TMJ can therefore be examined using different methods including panoramic radiography, open- and closed-mouth transcranial plain radiographs, conventional linear tomography, and computed tomography, which have been used to evaluate the bony joint components, while magnetic resonance imaging (MRI) is used to examine soft tissue components [2, 17]. Nowadays, cone-beam computed tomography (CBCT) enables the acquisition of high-resolution, multiplanar images with reduced radiation exposure [17]. These images offer accurate, undistorted measurements of joint structures and articular space, making CBCT the preferred noninvasive method for detecting bony TMJ variations [18, 19].

Considering that is quite important to evaluate the morphology, position, and dimensions of the articular structures in order to stablish theories concerning to the etiopathogenesis of the articular disturbances, optimize the planning of surgical procedures in the TMJ, determine the effects of the surgical approaches in this articulation, and prevent possible complications, this study aimed, using a multivariate binary logistic regression analysis, to identify demographic, morphologic, and morphometric variables that are independently associated with early or subclinical imaging-based osteoarthritic structural changes in the TMJ of asymptomatic patients. Additionally, it sought to determine which of these variables have a modifying effect on the occurrence of such changes.

## Materials and methods

### Setting, study design, and sample composition

This cross-sectional analytical study was evaluated and accepted by the Institutional Ethics Board of the Faculty of Dentistry, University of Antioquia (Approval N° 170–2023) and conducted in compliance with the ethical guidelines of the Helsinki Declaration. The analysis was based on CBCT scans originally obtained for dento-maxillofacial diagnostic indications such as dental/zygomatic implants, oral rehabilitation procedures, orthodontics, oral diseases, and maxillofacial surgery treatment planning purposes. Considering a reported prevalence of 46.27% of one or more osteoarthritic or degenerative changes in at least one of the TMJs [13], sample size calculation using a web-based calculator (https://www.calculator.net/sample-size-calculator.html), indicated that a sample of at least 271 TMJs would be necessary to determine significant differences between the groups with 90% confidence and a margin of error 5%. Nevertheless, a greater number of cases were included to preserve an optimal level of precision.

The sample was selected from patients underwent CBCT examination between January 2017 and April 2024. Written informed consent was obtained from the patients to use the images for study purposes prior to carrying out the CBCT scans. Clinical records and full-skull CBCT scans of patients without TMJ complaints of both sexes, aged ≥ 18 years, acquired in the position of maximum intercuspation, and oriented using the Frankfort horizontal and mid-facial planes as landmark, were considered for inclusion in the study. The age limit was selected owing to the occlusion and craniofacial skeleton, including the TMJ, undergo final maturation at about the age of 18 years [20]. In turn, the following exclusion criteria were considered: *1*) any previous diagnosis or management of temporomandibular joint disorders (TMD) and/or orofacial pain documented in the clinical records; *2)* CBCT scans obtained with open mouth; *3)* poor‑quality images, restricted fields of view, or artifacts that hindered measurement; *4)* cases revealing ankyloses of TMJ that preclude the measurement of joint structures; *5*) evidence of condylar hyperplasia or mandibular asymmetry; *6*) a history of surgical procedures, ongoing orthodontic treatment, traumatic injuries, as well as fibro-osseous, infectious or neoplastic diseases of the of the jaws, and syndromic craniofacial anomalies; and *7*) systemic diseases or use of medications known to affect joint structures.

### Image acquisition and standardization

Images were acquired using a Planmeca ProMax 3D Classic equipment (Planmeca^®^, Helsinki, Finland) set at 90 kVp, 8–14 mA, 13.8 s exposure time, 20.1 cm x 17.5 cm of field vision, and 400 μm voxel size. The images were assessed using the Romexis Viewer 5.3.4.39 software (Planmeca^®^) concurrently by two Oral and Maxillofacial Surgery Senior Residents (D. L-A. and L. G. L-B.) previously trained and standardized by two experienced Maxillofacial Radiologists (C. I. S-N. and J. G-G.). Standardization was performed with a set of reference images and written instructions concerning morphologic and morphometric features that would be evaluated until raters reached intraclass correlation coefficient (ICC), Cohen’s Kappa (κ) or weighted Kappa (κ_w_) values of ≥ 0.8. All observations were carried out in a darkened room adjusting the brightness and contrast to enhance image quality and the magnification function to amplify the image size.

### Patient-related data collection

**Fig. 1:**
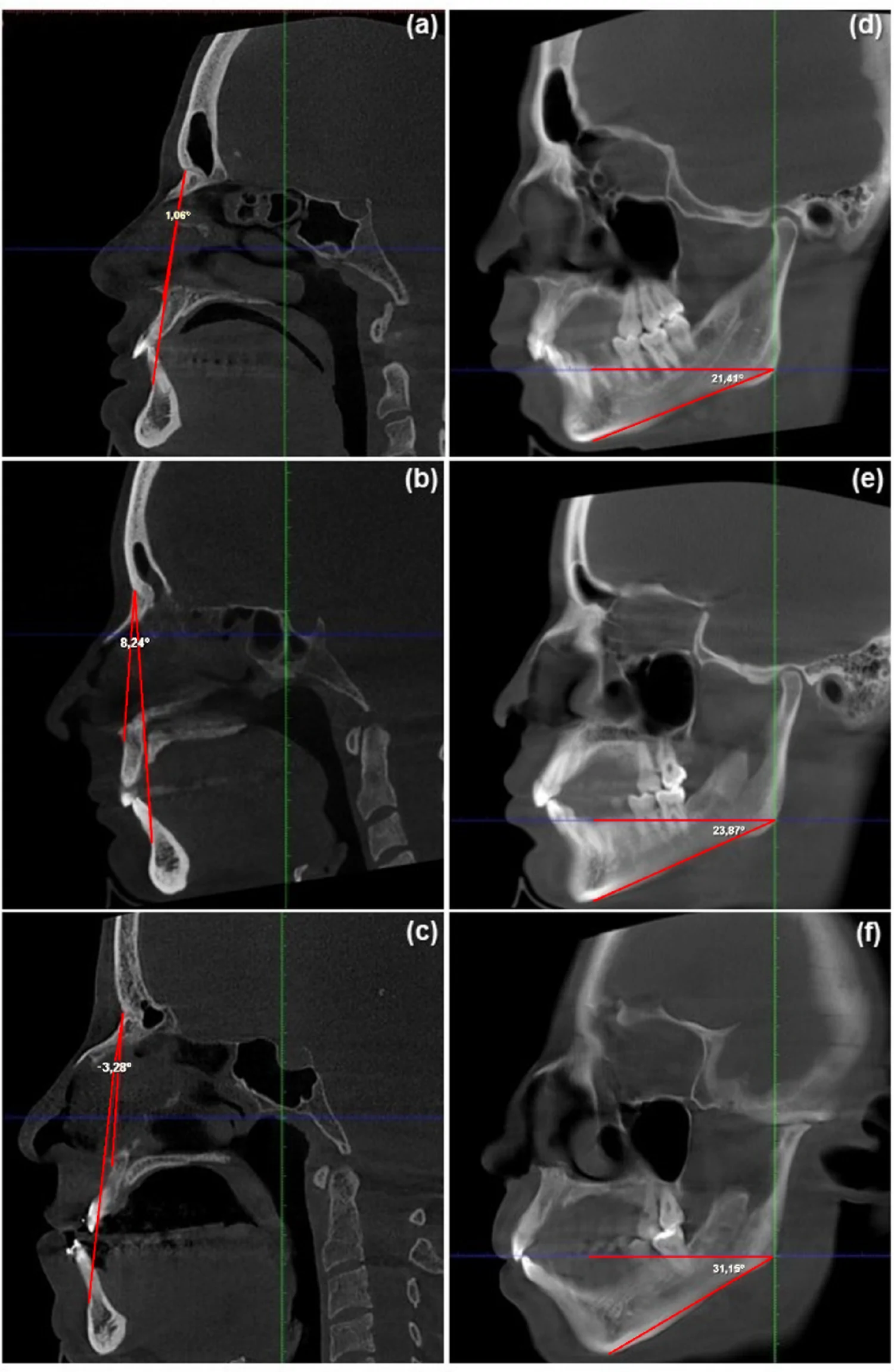
Sagittal CBCT reconstructions showing the angular measurements used to determine the anteroposterior maxillo-mandibular relationship and the vertical growth pattern. Based on the ANB angle the cases were characterized as **a** Class I, **b** Class II, or **c** Class III. According to the MP angle, the skeletal pattern was defined as **d** hypodivergent, **e** normodivergent, or **f** hyperdivergent. The lines were enhanced using the AxioVision 3.1^®^ (Carl Zeiss^®^, Oberkochen, Germany) software to illustrate the path by which the measurements were obtained

Data regarding study sample characteristics such as age of the patient at the time of CBCT acquisition and sex (i.e., *male vs. female*) were collected for each case. Also, the alveolar process status was documented and categorized based on the presence or absence of teeth as *fully dentate* or *completely/partially edentulous* (those with totally edentulous arches or at least one extracted tooth, except for third molars) [21]. In turn, based on the measurement of the angle formed by the point landmarks A, N, and B (ANB angle), the skeletal relationship was categorized as *Class I*, 0.1°≤ ANB ≤ 3.9°; *Class II*, ANB ≥ 4.0°; or *Class III*, ANB ≤ 0.0° (Fig. 1a-c) [22], whereas the vertical growth pattern was stratified on 5.2-mm-thick sagittal slices, using the mandibular plane (MP) angle, formed by the intersection between the Frankfort horizontal plane and the mandibular plane which connects the gonion (Go) and menton (Me) landmarks, as *hypodivergent*, MP ≤ 23°, *normodivergent*, 23°< MP < 30°, and *hyperdivergent*, MP ≥ 30° (Fig. 1d-f) [23].

## Assessment of TMJ structures

**Fig. 2:**
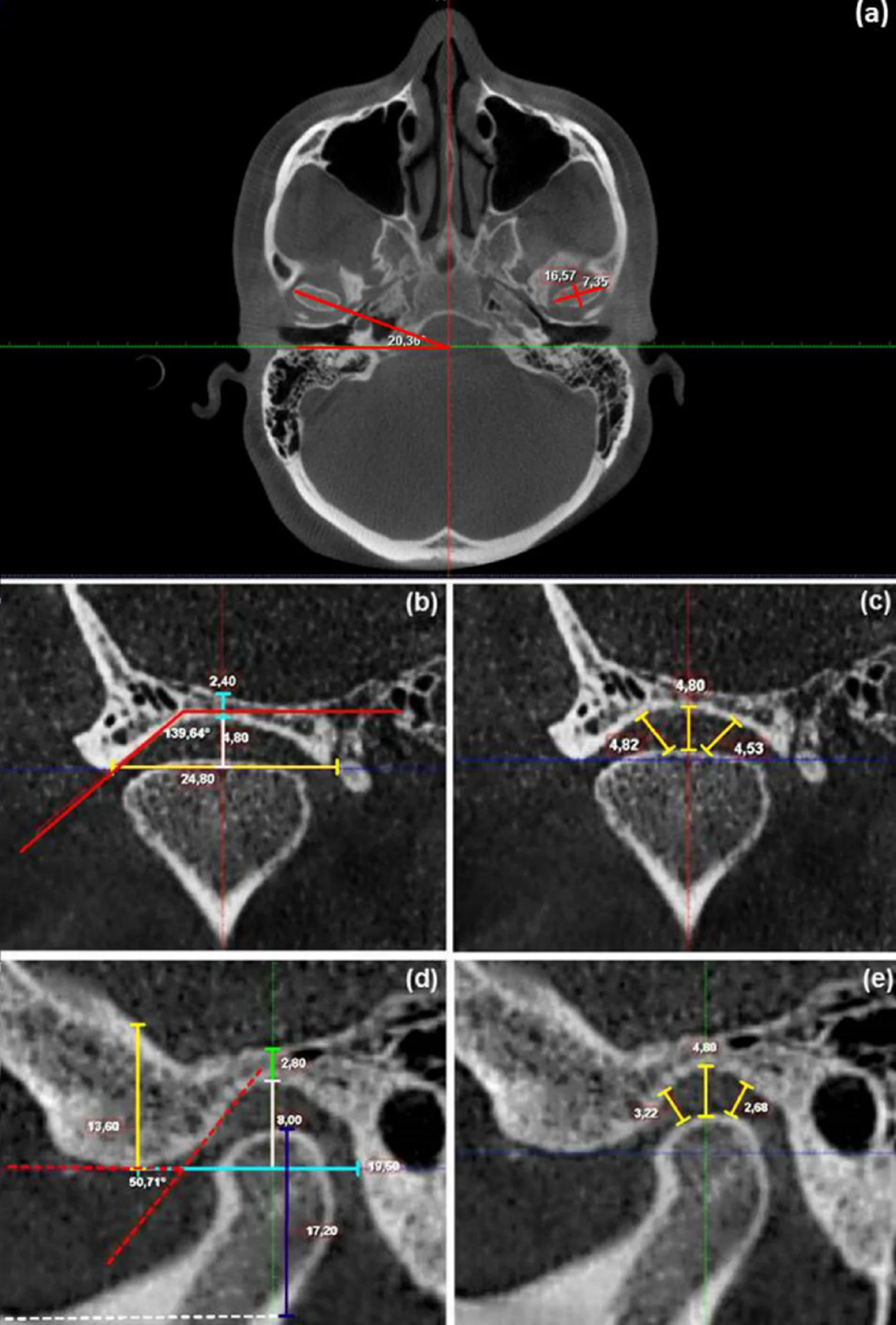
Typical CBCT images illustrating **a** the way of measuring the angulation of the horizontal condylar angle, as well as the mediolateral and anteroposterior condylar widths in the axial plane, **b** Coronal section illustrating the landmarks selected to determine the minimum glenoid fossa roof thickness (green line), the width (yellow line) and depth (white line) of the glenoid fossa, and the inclination of the lateral wall of the glenoid fossa (red lines). **c** Coronal view depicting the widths of the lateral and medial interarticular spaces measured at 45° from the central interarticular space (yellow lines). **d** Image demonstrating the method of measurement of some variables in the sagittal plane including: the condylar height (solid purple line), width (solid blue line) and depth (solid white line) of the glenoid fossa, minimum glenoid fossa roof thickness (solid green line), the height of the articular eminence (solid yellow line), and the inclination of the articular eminence (dotted red lines). Dotted white line represents the reference line to assist in the measurement of condylar height. **e** Sagittal view describing the widths of the anterior and posterior interarticular spaces measured at 30° from the central interarticular space (yellow lines). All of the lines were enhanced using the AxioVision 3.1^®^ software

The evaluation of the articular structures included the recording of morphological and dimensional variables and was carried out separately for each side using axial, coronal, and/or sagittal reconstructions following pre-established criteria according to each parameter, as described below:

- The horizontal condylar angle (i.e., the angle between the medio-lateral plane of the condylar process and a straight-line tangent to the posterior wall of external acoustic meatus [24], as well as the mediolateral and anteroposterior condylar widths (Fig. 2a), were traced at the condyle´s widest point in the axial plane [6].
- The morphometric measurements taken in the coronal plane (Fig. 2b) were: the minimum glenoid fossa roof thickness [5]; the width and depth of the glenoid fossa obtained through reference lines drawn between the petrosphenoidal fissure and the zygomatic process of the temporal bone, and from the roof of the glenoid fossa to the center of the former reference line, respectively [18, 25]; the inclination of the lateral wall of the glenoid fossa, defined as the angle formed by a line drawn along the inner wall of the zygomatic process of the temporal bone and the horizontal plane passing through the deepest point of the roof of the glenoid fossa [14]. Based on the same landmarks, the width of the central coronal space was measured from the roof of the glenoid fossa to the upper condyle border, while the widths of the lateral and medial spaces were calculated with an inclination of 45° from the central interarticular space (Fig. 2c).
- Dimensional parameters obtained in the sagittal plane (Fig. 2d) included: condylar height, defined as the perpendicular measurement from the condyle’s superior-most aspect of the condyle to a tangent drawn through the lower-most point of the sigmoid notch to the rear margin of the ramus [5]; the width and depth of the glenoid fossa were measured on the sagittal plane corresponding to the selected coronal view through reference lines drawn between the apex of the articular eminence and the posterior wall of the glenoid fossa, and from the roof of the glenoid fossa to the center of this reference line, respectively; the minimum glenoid fossa roof thickness; the height of the articular eminence defined as the perpendicular distance between the lower-most point on the articular eminence and the floor of the medial cranial fossa; the sagittal inclination of the articular eminence, defined as the angle formed by the tangent of the posterior surface of the articular eminence at the intersection point with the projection of the Frankfort plane. Also, the width of interarticular spaces in the sagittal plane (Fig. 2e) was calculated as described for the coronal view, but with an inclination of 30° from the central interarticular space [18].
- Alternatively, using selected measurements, four indexes were calculated as follows: *the coronal centering index* (CCI, the ratio between the widths of the lateral and medial interarticular spaces); *the sagittal centering index* (SCI, the ratio between the widths of the posterior and anterior interarticular spaces); *the lateral stability index* (LSI, the ratio between the width of the condyle and that of the glenoid fossa); and *the coverage index* (CI, the ratio among depth and width of the glenoid fossa) were measured according previously published data [18].

### Case definitions

**Fig. 3:**
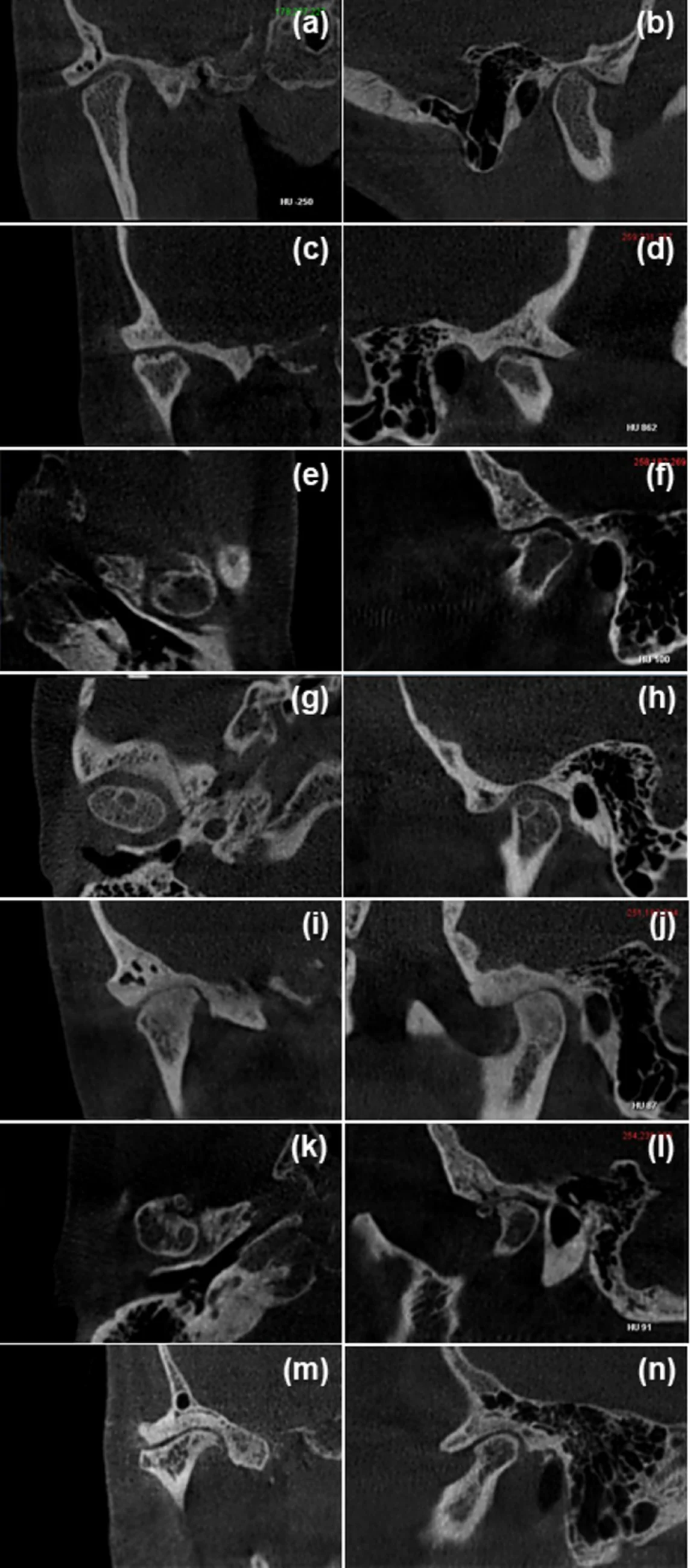
Illustrative images of bone changes detected in at least two concurrent tomographic projections: **a** coronal and **b** sagittal views showing a condylar flattening; **c** coronal and **d** sagittal cuts of a case of moderate erosion of the condylar surface; **e** axial and **f** sagittal slices demonstrating the presence of an osteophyte protruding from the anterolateral condylar surface; **g** axial and **h** sagittal views revealing the presence of a condylar subcortical cyst; **i** coronal and **j** sagittal pictures demonstrating generalized sclerosis of the articular structures; **k** axial and **l** sagittal cuts depicting intra-articular calcifications near the anterolateral condylar surface; **m** coronal and **n** sagittal cuts showing an extensive deviation in form of the condyle structure

The tomographic appearance of each TMJ was characterized in several conditions according to previous descriptions [10, 20, 26] as: *normal appearance*, absence of articular surface flattening, osteophytes, subcortical sclerosis, or subcortical cysts; *indeterminate for osteoarthritis (OA)*, isolated articular surface flattening or subcortical sclerosis; *affected by OA*, evidence of osteophytes, surface erosion, generalized sclerosis, or subcortical cysts. The morphologic changes considered in the assessments were: *flattening*, defined as a flat bony contour deviating from the convex form (Fig. 3a and b); *erosion*, featured by decreased density of the cortical bone and the adjacent subcortical bone (Fig. 3c and d); *osteophytes*, marginal bony outgrowths arising from the condyle surface (Fig. 3e and f); *subcortical cysts*, identified as a well-circumscribed hypodensities below the articular surface that deviates from normal marrow pattern (Fig. 3g and h); *sclerosis*, recognized as an area of increased density of cortical bone extending into the bone marrow (Fig. 3i and j); *intra-articular calcifications*, hyperdense zones located within articular spaces (Fig. 3k and l); and *deviation in form*, a variation from the normal condyle shape not attributable to the aforementioned morphologic changes (Fig. 3m and n) [10, 12, 15]. To avoid interpretation bias, bone changes should be found in at least two tomographic views. Each type of bony variation might be present alone or in combination with one or more additional alterations [15]. Moreover, the precise localization of the morphologic changes was specified for each finding (i.e., condyle, glenoid fossa, articular space, or combinations).

### Statistical data processing

Statistical analyses were carried out with SPSS Statistics 29.0 (IBM, Armonk, NY). If the same case had synchronously different TMJ conditions on both sides, the TMJ with the poorest condition was used as predominant diagnosis for the patient-level analysis. After a twelve-month study period, focused on performing the proposed observations, 17 TMJ scans (4.29% of the sample) was selected following a simple random sampling procedure, and reassessed synchronously by the same examiners to determine intra-observer variability using the Cohen’s κ or the κ_w_ statistics for categorical variables, and ICC for quantitative variables.

Bivariate analyses were achieved to detect between-groups differences regarding each evaluated parameter and to identify potential indicators for association with the indeterminate or the affected by OA classifications. The Kolmogorov-Smirnov test was used to assess the distribution of the quantitative variables. Considering that data were conformed to a normal distribution pattern, they were analyzed using the parametric one-way analysis of variance (ANOVA) with *post-hoc* Tukey’s *HSD* multiple comparison tests. Also, Pearson’s chi-square test (χ^2^) was used to compare categorical variables.

All significant predictor variables identified in the bivariate analyses were subjected to univariate and multivariate binary logistic regression analyses to confirm the association of potential indicators with the indeterminate or the affected by OA status whilst adjusting for confounding variables with *P*-values ≤ 0.20. With this purpose quantitative data included in the models were dichotomized according to the optimal cut-offs points obtained from receiver operating characteristic (ROC) curve analysis, whereas categorical variables were dichotomized according to clinically relevant distributions. Positive associations were considered when the odds ratio (OR) was greater than 1 and the confidence interval (CI) was > 1.0 on any of the constructs. Furthermore, the Hosmer-Lemeshow test was applied to evaluate the calibration accuracy of each multivariate model. Significance level was set at *P* < 0.05.

## Results

### Reliability analysis and sample composition

None of the individuals included in the study had present pain or TMJ complaints. A total of 367 CBCT scans were excluded for different reasons, including 155 scans that did not completely cover both TMJ, 74 symptomatic patients referred with previous diagnosis of TMD, 55 CBCT scans acquired in open-mouth position, 52 cases younger than 18 years of age, 26 cases with evidence of dentofacial trauma or orthognathic surgery, and 5 syndromic patients. In general, the data from repeated measurements obtained by the same examiners in the consistency analyses were dispersed around the mean or the observed values, with no trends towards over- or under-estimation, so that the outcomes did not reveal important differences for intra-observer reproducibility, neither for continuous nor for categorical variables evaluated. Therefore, the reliability values varied between 0.816 and 1.000, being significantly excellent (*P* < 0.001; ICC, Cohen’s κ, and κ_w_ tests) for all the variables. The sample involved 198 CBCT Digital Imaging and Communications in Medicine (DICOM) files showing both right and left TMJs (*n* = 396). The files were obtained from 75 males ranging in age from 18 to 85 years (mean 38.29 ± 16.23) and 123 females ranging in age from 18 to 86 years (mean 39.69 ± 16.19). Irrespective of diagnostic categories, descriptive analysis of the patient-level data showed that 139 patients (70.20%) were fully dentate, while 59 patients (29.80%) were categorized as completely/partially edentulous. It was also noteworthy that more than half of the patients presented a Class II skeletal relationship (101 cases, 51.00%), followed by class I (71 cases, 35.90%), and Class III (26 cases, 13.10%). Likewise, more than half of the patients (113 cases, 57.10%) had a hypodivergent growth pattern, followed by normodivergent (69 cases, 34.80%), and hyperdivergent (16 cases, 8.10%).

Based on TMJ-level data, it could be observed that 106 TMJs (26.80%) showed different combinations of osteoarthritic changes, followed by 74 TMJs (18.70%) with isolated articular surface flattening, and just a small proportion of isolated cases of erosion (6 TMJs, 1.50%), subcortical sclerosis (6 TMJs, 1.50%), osteophytes (3 TMJs, 0.80%), subcortical cysts (1 TMJ, 0.30%), and deviation in form (1 TMJ, 0.30%). Among the cases with combined alterations, 50 TMJs (47.20%) revealed up to two different types of osteoarthritic changes, and 56 TMJs (52.80%) showed three or more types of osteoarthritic changes. Most of the articular changes affected only the mandibular condyle (114 cases, 28.80%), followed by combined alterations of the articular structures (81 cases, 20.50%), and only two cases (0.50%) with isolated involvement of the glenoid fossa. Table 1 depicts the CBCT features used to define each diagnostic category. As expected, according to the case definitions, the normal-appearing TMJs exhibited no CBCT changes in any of the joint structures, whereas indeterminate cases had different proportions of flattening, subcortical sclerosis, or subtle deviation in form; and the cases affected by OA showed, in addition to the above changes, evidence of erosion, osteophytes, and subcortical cysts. From these TMJ-level data it was defined that 199 TMJs (50.30%) presented normal appearance, whereas 100 TMJs (25.30%) had a diagnosis of indeterminate for OA, and 97 TMJs (24.50%) were affected by OA.

**Table 1:** Summary of CBCT findings obtained from the study sample used to determine the diagnostic category

CBCT findings		Diagnostic category^a^		
		Normal-appearing group (*n* = 199)	Indeterminate for OA group (*n* = 100)	Affected by OA group (*n* = 97)
Flattening	Present	--	95 (95.00)	75 (77.30)
	Absent	199 (100.00)	5 (5.00)	22 (22.70)
Erosion	Present	--	--	57 (58.80)^b^
	Absent	199 (100.00)	100 (100.00)	40 (41.20)
Osteophytes	Present	--	--	52 (53.60)
	Absent	199 (100.00)	100 (100.00)	45 (46.40)
Subcortical cysts	Present	--	--	22 (22.70)
	Absent	199 (100.00)	100 (100.00)	75 (77.30)
Subcortical sclerosis	Present	--	19 (19.00)	42 (43.30)
	Absent	199 (100.00)	81 (81.00)	55 (56.70)
Intra-articular calcifications	Present	--	--	22 (22.70)
	Absent	199 (100.00)	100 (100.00)	75 (77.30)
Deviation in form	Present	--	10 (10.00)	32 (33.00)
	Absent	199 (100.00)	90 (90.00)	65 (67.00)

^a^Values are given as*n* (%) of cases within each parameter according the diagnostic category

^b^Including 31 cases (54.40%) with slight erosion, 18 cases (31.60%) with moderate erosion, and 8 cases (14.00%) with extensive erosion

The information obtained at patient-level revealed that 81 cases (40.90%) showed bilateral normal-appearing TMJs, and 78 cases (39.30%) showed different bilateral abnormalities, including 29 cases (14.60%) bilaterally affected by OA, 28 cases (14.10%) bilaterally classified as indeterminate for OA, and 21 (10.60%) cases affected by bilateral combinations of indeterminate/osteoarthritic changes. Also, 39 cases (19.70%) showed unilateral normal-appearing TMJ combined with contralateral indeterminate/osteoarthritic changes, including 23 cases (11.6%) of unilateral indeterminate presentations, and 16 cases (8.10%) with unilateral osteoarthritic changes. Considering the poorest condition in patient-level analyses, the prevalence rates revealed that 40.90% (81 patients) presented normal appearance, 25.80% (51 patients) received a diagnosis of indeterminate for OA, and 33.30% (66 patients) were affected by OA.

### Outcomes of bivariate comparisons

Bivariate comparisons of the parameters assessed at the patient-level with reference to the predominant tomographic TMJ appearance are outlined in Table 2. Patients classified predominantly as indeterminate for OA only exhibited a significantly higher frequency of complete/partial edentulism (*P* < 0.05, χ² *post hoc* pairwise-comparison test) compared with those with normal TMJ appearance. Likewise, the patients diagnosed predominantly as affected by OA had a significantly greater mean age and a significantly higher prevalence of complete/partial edentulism in comparison to the normal-appearing patients (*P* < 0.05, Tukey *HSD* and χ^2^ *post hoc* pairwise-comparison tests). In addition, this later group also showed a significantly greater proportion of Class II skeletal relationship in comparison to the normal-appearing and indeterminate counterparts (χ^2^ *post hoc* pairwise-comparison tests). Therefore, those variables were considered candidate risk indicators to be included in the multivariate models. Conversely, there were no significant differences (*P* > 0.05, χ^2^ test) among the study groups regarding to the growth pattern. Nevertheless, sex exerted a confounding influence on the results, as these indicators reached a *P*-value < 0.20.

**Table 2:** Resume of patient-level characteristics obtained from the study sample with reference to the tomographic TMJ appearance

Parameter		Tomographic TMJ appearance			*P*-value
		Normal-appearing group (*n* = 81)	Indeterminate for OA group (*n* = 51)	Affected by OA group (*n* = 66)	
Age (years)^a^		35.84 ± 14.97	39.88 ± 16.65	42.68 ± 16.67^*^	0.028^d^
Sex^b^	Male	34 (42.00)	23 (45.10)	18 (27.30)	0.088^e^
	Female	47 (58.00)	28 (54.90)	48 (72.70)	
Alveolar process status^b^	Fully dentate	65 (80.20)	32 (62.70)	41 (62.10)	0.027^e^
	Completely/partially edentulous^c^	16 (19.80)	19 (37.30)^†^	25 (37.90)^†^	
Skeletal relationship^b^	Class I (0.1°≤ ANB ≤ 3.9°)	36 (44.00)	21 (41.20)	14 (21.20)	< 0.001^e^
	Class II (ANB ≥ 4.0°)	28 (34.60)	24 (47.10)	49 (74.20)^†‡^	
	Class III (ANB ≤ 0.0°)	17 (21.00)	6 (11.80)	3 (4.50)	
Vertical growth pattern^b^	Hypodivergent (MP ≤ 23°)	50 (61.70)	31 (60.80)	32 (48.50)	0.516^e^
	Normodivergent (23°< MP < 30°)	25 (30.90)	17 (33.30)	27 (40.90)	
	Hyperdivergent (MP ≥ 30°)	6 (7.40)	3 (5.90)	7 (10.60)	

^a^Values given as mean ± SD

^b^Values given as n (%) of patients within each parameter according to the tomographic TMJ appearance

^c^Including 40 cases (23.00%) with posterior edentulous areas and 17 cases (9.80%) with combined anterior/posterior edentulous areas

^d^One-way ANOVA

*Statistically significant difference (*P* < 0.05, Tukey *HSD* multiple comparison test) as compared with normal appearance group

^e^Two-sided Pearson χ^2^ test

^†^Statistically significant difference (*P* < 0.05, χ^2^ post-hoc comparison test) as compared with normal appearance group

^‡^Statistically significant difference (*P* < 0.05, χ^2^ post-hoc comparison tests) in comparison with the indeterminate for OA group

On the other hand, between-group comparisons of candidate risk indicators in relation to diagnostic categories at the TMJ-level are presented in Table 3. Regarding categorical variables, this table clearly shows that although no significant differences were observed among study groups for the sagittal centering, lateral stability, and coverage indexes (*P* > 0.05, χ^2^ test), both the involved side as well as the coronal centering index had a confounding influence on the results (*P* < 0.20). As also can be seen from this table, while no significant differences (all *P* > 0.05 1-way ANOVA test) were detected between the study groups with respect to continuous variables such as anteroposterior condylar width at axial plane; height of articular eminence at sagittal plane; inclination of the lateral wall of the glenoid fossa, medial interarticular space at coronal plane; nor for central, anterior, and posterior interarticular spaces at sagittal plane; those data regarding condylar height, angle of the articular eminence at sagittal plane, and lateral interarticular space at coronal plane were significantly lower (all *P* < 0.05, 1-way ANOVA/Tukey *HSD post hoc* multiple-comparison tests) in the affected by OA group in comparison with the normal-appearing group. Likewise, the mean value of mediolateral condylar width at axial plane was significantly lower for the affected by OA group compared to the other two groups (*P* < 0.05). Conversely, the mean values of the horizontal condylar angle, width of glenoid fossa at sagittal plane, as well as the glenoid fossa roof thickness at coronal and sagittal planes were significantly greater (*P* < 0.05) in the affected by OA group in comparison with the normal-appearing group. In contrast, the width and depth of glenoid fossa at coronal plane, the depth of glenoid fossa at sagittal plane, and the central interarticular space at coronal plane, also fulfilled the condition to be deemed as independent confounding variables for the association between candidate risk variables and the diagnostic categories (*P* < 0.20). It was also remarkable that none of the variables under evaluation showed significant differences between the normal-appearing and indeterminate groups in the bivariate comparisons performed at the TMJ-level. Since comparing the variables that differentiate these two groups would not yield significant predictors of the outcome (i.e., all parameters would show OR values < 1 and IC values ≤ 1), those cases diagnosed as indeterminate for OA were excluded from logistic regression analyses at the TMJ-level.

**Table 3:** Comparison of candidate risk variables gathered at TMJ-level with reference to the diagnostic categories

Parameter			Tomographic TMJ appearance				*P*-value
			Normal-appearing group (*n* = 199)		Indeterminate for OA group (*n* = 100)	Affected by OA group (*n* = 97)	
Side^a^	Right		104 (52.30)		40 (40.00)	54 (55.70)	0.059^c^
	Left		95 (47.70)		60 (60.00)	43 (44.30)	
Horizontal condylar angle^b^			21.83 ± 9.74		23.92 ± 9.73	27.33 ± 11.67^†^	< 0.001^d^
Mediolateral condylar width at axial plane^b^			17.44 ± 2.50		17.63 ± 2.45	16.46 ± 2.14^**†‡**^	0.001^d^
Anteroposterior condylar width at axial plane^b^			7.34 ± 1.30		7.30 ± 1.37	7.34 ± 1.71	0.965^d^
Condylar height^b^			16.63 ± 2.39		16.35 ± 2.72	15.65 ± 2.18^**†**^	0.006^d^
Width of glenoid fossa at coronal plane^b^			21.77 ± 2.20		21.92 ± 1.77	21.24 ± 2.26	0.055^d^
Depth of glenoid fossa at coronal plane^b^			5.74 ± 1.21		5.75 ± 1.22	5.40 ± 1.38	0.067^d^
Width of glenoid fossa at sagittal plane^b^			16.74 ± 2.47		16.97 ± 2.62	17.84 ± 2.90^**†**^	0.003^d^
Depth of glenoid fossa at sagittal plane^b^			6.50 ± 1.55		6.43 ± 1.47	6.94 ± 2.22	0.074^d^
Height of articular eminence at sagittal plane^b^			8.57 ± 2.68		8.55 ± 2.30	8.93 ± 2.55	0.467^d^
Angle of the articular eminence at sagittal plane^b^			47.36 ± 10.58		47.38 ± 10.94	43.87 ± 11.54^**†**^	0.024^d^
Inclination of the lateral wall of the glenoid fossa^b^			143.54 ± 10.68		143.73 ± 9.73	143.09 ± 11.55	0.908^d^
Glenoid fossa roof thickness at coronal plane^b^			1.30 ± 0.66		1.40 ± 0.87	1.64 ± 1.12^†^	0.006^d^
Glenoid fossa roof thickness at sagittal plane^b^			1.18 ± 0.52		1.28 ± 0.82	1.51 ± 1.11^†^	0.003^d^
Central interarticular space at coronal plane^b^			2.63 ± 0.94		2.61 ± 1.06	2.35 ± 1.01	0.059^d^
Lateral interarticular space at coronal plane^b^			2.57 ± 0.98		2.34 ± 0.96	2.10 ± 0.71^†^	< 0.001^d^
Medial interarticular space at coronal plane^b^			2.85 ± 1.05		2.93 ± 1.08	2.70 ± 1.36	0.373^d^
Central interarticular space at sagittal plane^b^			2.58 ± 0.93		2.51 ± 1.08	2.38 ± 1.02	0.256^d^
Anterior interarticular space at sagittal plane^b^			2.46 ± 0.82		2.60 ± 0.97	2.36 ± 1.14	0.218^d^
Posterior interarticular space at sagittal plane^b^			2.54 ± 1.12		2.37 ± 1.10	2.46 ± 1.08	0.481^d^
Coronal centering index^a^		Centered (CCI = 1)		31 (15.60)	11 (11.00)	7 (7.20)	0.154^c^
		Medially centered (CCI > 1)		63 (31.70)	28 (28.00)	26 (26.80)	
		Laterally centered (CCI < 1)		105 (52.70)	61 (61.00)	64 (66.00)	
Sagittal centering index^a^		Centered (SCI = 0.9)		7 (3.50)	5 (5.00)	3 (3.10)	0.426^c^
		Anteriorly centered (SCI > 0.9)		112 (56.30)	45 (45.00)	54 (55.70)	
		Posteriorly centered (SCI < 0.9)		80 (40.20)	50 (50.00)	40 (41.20)	
Lateral stability index^a^		Stable (LSI ≤ 0.85)		138 (69.30)	74 (74.00)	76 (78.40)	0.250^c^
		Unstable (LSI > 0.85)		61 (30.70)	26 (26.00)	21 (21.60)	
Coverage index^a^		Constrained (CI ≥ 0.20)		180 (90.50)	89 (89.00)	82 (84.50)	0.319^c^
		Unconstrained (CI < 0.20)		19 (9.50)	11 (11.00)	15 (15.50)	

^a^Values given as n (%) of TMJs within each parameter according to the tomographic appearance

^b^Values given as mean ± SD

^c^Two-sided Pearson χ^2^ test

^*^Statistically significant difference (*P* < 0.05, χ^2^ post-hoc comparison test) as compared with normal appearance group

^e^One-way ANOVA

^†^Statistically significant difference (*P* < 0.05, Tukey *HSD* multiple comparison test) as compared with normal appearance group

^‡^Statistically significant difference (*P* < 0.05, χ^2^ post-hoc comparison tests) in comparison with the indeterminate for osteoarthritis group

### Outcomes from univariate and multivariate logistic regression analyses

Regression models estimating the risk associated with candidate indicators for the diagnosis categories are presented in Tables 4 and 5. These models, evaluated at both the patient- and TMJ-levels, are shown before and after adjustment for confounding variables identified through bivariate analyses. Overall, the values of Hosmer-Lemeshow goodness-of-fit test fluctuated between 0.062 and 0.990 confirming a good calibration and fit of the regression models both at patient- and TMJ-level. As can be seen from Table 4, at the patient-level, the OR for the indeterminate group was significantly increased (*P* < 0.05, Wald’s test) in individuals completely/partially edentulous. In addition, the OR for the affected by OA group was significantly increased (*P* < 0.05) in individuals with age > 38.50 years, completely/partially edentulous, and with Class II skeletal relationship. After adjusting for sex as confounder, all these candidate risk indicators remained sturdily associated with the diagnosis of either indeterminate or affected by OA (*P* < 0.05).

**Table 4:** Summary of univariate and multivariate binary logistic regression analyses for the association of candidate risk indicators with the diagnosis of indeterminate for OA and affected by OA at the patient-level after adjusting for sex

Candidate risk indicators			Cases^a^		Univariate analysis		Multivariate binary logistic regression analysis		Calibration^d^
			Normal-appearing group	Affected by OA group	Unadjusted OR (95% CI)^b^	*P*-value^c^	Adjusted OR (95% CI)^b^	*P*-value^c^	
*Indeterminate for OA*									
Alveolar process status		Fully dentate	65 (80.20)	32 (62.70)	Referent	0.029		0.033	0.471
		Completely/partially edentulous	16 (19.80)	19 (37.30)	2.41 (1.10–5.31)		2.38 (1.07–5.29)		
*Affected by OA group*									
Age^e^	≤ 38.50 years		51 (63.00)	27 (40.90)	Referent	0.008		0.008	0.990
	> 38.50 years		30 (37.00)	39 (59.10)	2.46 (1.26–4.78)		2.46 (1.26–4.79)		
Alveolar process status	Fully dentate		65 (80.20)	41 (62.10)	Referent	0.016		0.016	0.961
	Completely/partially edentulous		16 (19.80)	25 (37.90)	2.48 (1.18–5.20)		2.50 (1.19–5.26)		
Skeletal relationship	Class I/Class III		53 (65.40)	17 (25.80)	Referent	< 0.001		< 0.001	0.929
	Class II		28 (34.60)	49 (74.20)	5.46 (2.66–11.18)		5.50 (2.68–11.30)		

^a^Values are given as n (%) of patients within diagnosis groups

^b^Odds ratio (95% confidence interval)

^c^Wald test

^d^Hosmer & Lemeshow goodness-of-fit test

^e^Values dichotomized according ROC curve analysis

**Table 5:** Summary of univariate and multivariate binary logistic regression analyses for the association of candidate risk indicators with the diagnosis of affected by OA at the TMJ-level after adjusting for confounding covariates^a^

Candidate risk indicators		Cases^b^		Univariate analysis		Multivariate binary logistic regression analysis		Calibration^e^
		Normal-appearing group	Affected by OA group	Unadjusted OR (95% CI)^c^	*P*-value^d^	Adjusted OR (95% CI)^c^	*P*-value^d^	
Horizontal condylar angle^f^	< 24.14 degrees	116 (58.30)	37 (38.10)	Referent	0.001		0.006	0.375
	≥ 24.14 degrees	83 (41.70)	60 (61.90)	2.27 (1.38–3.72)		2.07 (1.24–3.47)		
Mediolateral condylar width at axial plane^f^	> 17.08 mm	108 (54.30)	39 (40.20)	Referent	0.024		0.073	0.062
	≤ 17.08 mm	91 (45.70)	58 (59.80)	1.77 (1.08–2.89)		1.68 (0.95–2.97)		
Condylar height^f^	> 16.00 mm	116 (58.30)	39 (40.20)	Referent	0.004		0.024	0.741
	≤ 16.00 mm	83 (41.70)	58 (59.80)	2.08 (1.27–3.41)		1.83 (1.08–3.08)		
Width of glenoid fossa at sagittal plane^f^	≤ 16.95 mm	99 (47.70)	35 (36.10)	Referent	0.024		0.023	0.478
	> 16.95 mm	100 (50.30)	62 (63.90)	1.75 (1.06–2.89)		1.88 (1.09–3.22)		
Angle of the articular eminence at sagittal plane^f^	> 45.78 degrees	108 (54.30)	38 (39.20)	Referent	0.015		0.002	0.268
	≤ 45.78 degrees	91 (45.70)	59 (60.80)	1.84 (1.12–3.02)		2.54 (1.40–4.60)		
Glenoid fossa roof thickness at coronal plane^f^	≤ 1.23 mm	127 (63.80)	43 (44.30)	Referent	0.002		0.001	0.448
	> 1.23 mm	72 (36.20)	54 (55.70)	2.22 (1.35–3.63)		2.38 (1.41–4.01)		
Glenoid fossa roof thickness at sagittal plane^f^	≤ 1.20 mm	141 (70.90)	47 (48.50)	Referent	< 0.001		< 0.001	0.191
	> 1.20 mm	58 (29.10)	50 (51.50)	2.59 (1.57–4.27)		2.95 (1.72–5.00)		
Lateral interarticular space at coronal plane^f^	> 2.25 mm	121 (60.80)	46 (47.40)	Referent	0.030		0.599	0.452
	≤ 2.25 mm	78 (39.20)	51 (52.60)	1.72 (1.05–2.81)		1.18 (0.64–2.15)		

^a^Including involved side, width and depth of glenoid fossa at coronal plane, depth of glenoid fossa at sagittal plane, central interarticular space at coronal plane, and coronal centering index

^b^Values are given as n (%) of TMJs within diagnosis groups

^c^Odds ratio (95% confidence interval)

^d^Wald test

^e^Hosmer & Lemeshow goodness-of-fit test

^f^Values dichotomized according ROC curve analysis

In the univariate analyses summarized in Table 5, several morphometric parameters demonstrated significant associations (*P* < 0.05) with the diagnosis of affected by OA at the TMJ level. Specifically, increased OR was observed in cases presenting with a horizontal condylar angle ≥ 24.14 degrees, mediolateral condylar width at the axial plane ≤ 17.08 mm, condylar height ≤ 16.00 mm, glenoid fossa width at the sagittal plane > 16.95 mm, articular eminence angle ≤ 45.78 degrees, glenoid fossa roof thickness at the coronal plane > 1.23 mm, glenoid fossa roof thickness at the sagittal plane > 1.20 mm, and lateral interarticular space at the coronal plane ≤ 2.25 mm. Following adjustment for confounding variables, the associations involving mediolateral condylar width ≤ 17.08 mm and lateral interarticular space ≤ 2.25 mm were attenuated and no longer statistically significant (*P* > 0.05), indicating the presence of confounding effects. Conversely, the associations for horizontal condylar angle ≥ 24.14 degrees, condylar height ≤ 16.00 mm, glenoid fossa width at sagittal plane > 16.95 mm, articular eminence angle at sagittal plane ≤ 45.78 degrees, and increased roof thickness of the glenoid fossa at both coronal (> 1.23 mm) and sagittal (> 1.20 mm) planes remained robust, independent, and statistically significant (*P* < 0.05). No evidence of interaction effects was detected among the covariates subject to confounding.

## Discussion

It is widely acknowledged that the etiology of TMD is complex and multifactorial, although its etiopathogenesis has not been completely understood. Given the variability of predisposing, initiating, and perpetuating factors, the treatment of TMD patients constitutes a clinical challenge [27], so that the research to understand the mechanistic basis of the cause-effect relationships requires special consideration. Although alterations in the osseous components of the temporomandibular joint may serve as indicators of degenerative processes that have recently arisen or are ongoing [20], it must be emphasized that such structural changes do not invariably correlate with clinical manifestations [28]. Accordingly, the present study employed full-skull CBCT scans to determine which demographic, morphologic, and morphometric variables are independently associated with, and which exert a modifying influence on, the occurrence of imaging-based osteoarthritic changes in asymptomatic TMJs. In this context, a deliberate decision was made to include only asymptomatic individuals, as this ensured that the morphologic and morphometric variations observed were not confounded by pain-related behaviors, prior diagnoses, or therapeutic interventions. By focusing on subjects free of TMJ symptoms, the study was able to isolate structural changes that may represent early or subclinical stages of osteoarthritis, thereby strengthening the reliability of the identified predictors and underscoring their potential value for risk assessment and monitoring in populations that might otherwise remain undiagnosed. It is important to underline that, since intra-raters’ agreement was excellent for all the parameters measured through multiplanar reconstructions, the herein results highlight that the detailed CBCT analysis of TMJ constitutes a valid, reliable, simple, and useful method in the clinical context. In addition, the morphometric predictors identified in this study may offer practical utility in clinical settings by supporting early identification of patients at risk for TMJ osteoarthritic changes. These anatomical features, readily assessable through CBCT imaging, could enhance risk stratification, guide targeted imaging protocols, and inform personalized management strategies such as occlusal adjustments, physiotherapy, or behavioral interventions. Moreover, visualizing these structural risk indicators may improve patient understanding and engagement with treatment plans. While further validation in longitudinal cohorts is needed, these findings suggest that morphometric analysis could serve as a valuable adjunct to clinical assessment, bridging the gap between imaging data and individualized care in TMD management [29].

Morphological changes in the TMJ bone components that accompany TMD may suggest remodeling processes and do not necessarily indicate disease progression [13]. Even so, several authors have demonstrated that joint bone abnormalities are significantly more prevalent in TMD patients [4, 30] and there is a consensus that imaging represents the reference standard for the diagnosis of degenerative joint disease [31, 32]. In this work, the findings from the CBCT images yielded a diagnosis of indeterminate for OA in 100 TMJs (25.30%) and affected by OA in 97 TMJs (24.50%). Other authors have reported, however, different diagnosis rates for the TMJ appearance in asymptomatic patients [10, 20], indicating that while there may be wide inter-individual variation [20], CBCT scans of asymptomatic TMJ patients regularly show degenerative bony alterations [33, 34]. Furthermore, considering that one TMJ can affect the other, it is also relevant to synchronously evaluate the tomographic appearance of the contralateral side [35]. Accordingly, in this study only 40.90% of the cases had bilateral normal-appearing TMJs, 39.30% of cases showed bilateral involvement, and 19.70% had some unilateral affectation, meaning that morphological changes were present in at least one joint in 117 individuals (59.00%). Although there is little information related to the bilateral and unilateral TMJ osteoarthritic changes on which a basis of comparison can be established, the present results contrast with those of an earlier work [35] which reported lower frequencies of bilateral involvement and higher frequencies of unilateral involvement in symptomatic patients. In contrast, this result concurs, at least partially, with that of a previous study [13] which showed that morphologic changes were present in at least one joint in 64.93% of asymptomatic individuals. A possible reason for all discrepancies in rates might lie in differences in the sample size and composition. Despite this study did not include information related to parafunctional activity and masticatory side preferences, it could be argued that an unbalanced distribution of biomechanical stress due to unilateral masticatory predominance may be a relatively consistent phenomenon linked to this finding [36].

Among patient-related indicators, age > 38.50 years and a Class II skeletal relationship were significantly associated with the likelihood of being diagnosed as affected by OA in both univariate and multivariate analyses, and these associations remained robust after adjustment for sex. In addition, complete or partial edentulism emerged as a strong and independent risk indicator, showing consistent associations with both the indeterminate and affected by OA diagnostic categories. As for age, some researchers have established, in concordance with the current results, that progression and severity of osseous changes in the condylar head and glenoid fossa can increase with ageing, mainly manifesting in the fourth or fifth decades of life [5, 9, 11, 12, 18, 35, 36]. In this study, age was categorized based on ROC curve analysis to identify the threshold that best discriminated between individuals with and without TMJ osteoarthritic changes. While this threshold improves interpretability in the regression modeling, it may also reflect a biologically or clinically meaningful transition point for the onset of degenerative changes or shifts in joint remodeling dynamics. Furthermore, individuals above 38.50 years may exhibit higher prevalence or severity of osteoarthritic changes, justifying age-based screening or preventive strategies. Although some of these changes may occur because of remodeling mechanisms or physiological responses to aging without clinical signs [37], others can be related to a reduced remodeling capacity of the TMJ fibrocartilage [38] together with a low condylar bone quality yielding to a thinner and damaged cortical bone and a lower trabecular bone density [39]. In contrast, several studies have not found an association between ageing and an increase in bone changes [10, 32, 33]. It is possible that the divergence between results may be related to differences the sample sizes and age distribution of the cases across the studies. In regard the effect of the skeletal relationship, it has been recognized that different skeletal patterns induce dissimilar stress distributions on the TMJ during mastication [16], so that the effect of skeletal pattern on its structure has long been a topic of scientific debate [20]. In this study, the cases affected by osteoarthritis showed a significantly greater proportion of Class II skeletal relationship in comparison to the normal-appearing and indeterminate groups. Although both divergent and similar data have been published [10, 20], in agreement with the current result, it has been demonstrated that the direction of the force vector on the condyle in Class II profiles is significantly greater than those of Class I and III [40]. In this way, the adjustments in the biophysical environment of the TMJ, derived from an altered mandibular positioning, can trigger the release of regulatory factors that promote dysfunctional remodeling of the morphology of the condyle and articular fossa [10, 20] leading to the development of osteoarthritic changes. Respecting alveolar process status, in opposition with some authors [41], edentulousness reached significantly different proportions between the tomographic TMJ appearance groups, thus representing a robust indicator for both indeterminate and affected by OA categories. Other authors coincide in pointing out that loss of teeth can affect bone structure, leading to a higher degree of degenerative bone changes [11, 36, 42, 43] possibly due to local mechanical factors encompassing parafunctional habits, functional overloading, and increased joint friction [11]. This finding reinforces the concept that edentulousness not only predisposes to generalized osteoarthritic alterations but also plays a direct role in condylar flattening, the most prevalent hallmark among individuals classified as indeterminate, likely as an early adaptive response to altered biomechanics. Therefore, condyle flattening in edentulous individuals should be interpreted as a critical morphological marker of compromised joint function, bridging the continuum between adaptive remodeling and overt osteoarthritic degeneration.

Accurate assessment of morphometric characteristics is critical for diagnosing osteoarthritic changes in TMJ bone components. In this study, the analysis at the TMJ-level revealed that although eight morphometric parameters were positively associated with the proportion of cases diagnosed as affected by OA in the bivariate and the univariate analyses, only six of these associations remained as strong and independent indicators after adjustment for confounder variables. Collectively, these indicators might be particularly important insofar as they disrupt the normal biomechanics of the TMJ, affecting how the condyle moves within the fossa. The increased risk observed with horizontal condylar angles ≥ 24.14 degrees is consistent with previous studies demonstrating that moderate to severe degenerative changes in TMJ are associated with a greater horizontal condylar angle due to a resorption process occurring at the anterolateral pole and bone formation at the posteromedial pole in the axial plane [24, 44, 45]. Notably, this threshold concurs with earlier findings linking angles in the 20–25° range to osteoarthritic changes, particularly in the context of condylar adaptation and remodeling [24, 45]. In turn, in this study, condylar height ≤ 16.00 mm was independently associated with the affected by OA group, underscoring the structural resorption and vertical shortening that accompany degenerative joint disease. Such reduction has been linked to flattening, erosion, and cortical loss [5, 10, 16, 20, 26, 32], which might compromise the condyle’s ability to distribute functional loads evenly across the articular surfaces. The resulting biomechanical imbalance may increase stress concentration within the glenoid fossa and articular eminence, perpetuating further degenerative changes and altering mandibular kinematics [46, 47]. Additionally, the robust association of the width of glenoid fossa at sagittal plane > 16.95 mm with the diagnosis of affected by OA is roughly in line with a previous work [48] indicating that in response to altered loading and stress distribution due to degenerative changes, the glenoid fossa may widen to accommodate changes in the condylar shape and movement. With reference to the angle of the articular eminence at sagittal plane ≤ 45.78 degrees, in consonance with the present results, it has been stated that the condyle and articular eminence morphology affect one another and are affected by other factors [8]. As the articular cartilage deteriorates, the condyle’s movement across the articular eminence becomes less smooth, so that abnormal forces and friction are generated between the joint surfaces. These altered forces can lead to remodeling of the bone and the flattening of the articular eminence slope [49]. It was also striking that the increased glenoid fossa roof thickness, > 1.23 mm at coronal plane and > 1.20 mm at sagittal plane showed a sturdy association with the diagnosis of affected by OA. These measurements closely approximate to previously published data (~ 0.9–1.2 mm) [5, 50], suggesting that osteoarthritic changes may influence glenoid fossa roof thickness by mechanical stimulation and changed stress distribution. Given that degenerative joint disease is often accompanied by chronic inflammation, inflammatory cytokines can stimulate osteoblastic activity, leading to a reactive bone formation thickening of the glenoid fossa roof. This thickening can be viewed as a protective mechanism, an attempt to reinforce the bone against the abnormal forces. It is also important to highlight that a cofounding effect was significantly detected for the mediolateral condylar width at axial plane ≤ 17.08 mm and the lateral interarticular space at coronal plane ≤ 2.25 mm. In the context of TMJ OA, this finding might indicate that the combined effect of these anatomical measurements is interdependent, meaning the effect of one factor depends on the magnitude of the other factors.

In this study, which exclusively included asymptomatic individuals, the presence of isolated changes of flattening, subcortical sclerosis, or deviation in form in cases categorized as indeterminate for OA, combined with the absence of significant differences in any of the evaluated indicators at TMJ-level in comparison with the normal appearance group, reinforces the notion that such findings may reflect physiological remodeling, adaptive responses to functional loading [10, 20, 33], or normal anatomical variation rather than early pathological changes. Nonetheless, indeterminate CBCT findings do not preclude the potential for future dysfunction, underscoring the importance of longitudinal monitoring in select cases [10].

Potential limitations of this study included: firstly, due to its observational nature and cross-sectional design, this study cannot explain longitudinal alterations in the bone structures nor establish a causal link between TMJ bone changes and OA. Secondly, the investigation did not assess symptomatic patients diagnosed with TMD, nor did it analyze their chewing patterns. Consequently, further prospective studies comparing TMD and non-TMD patients, considering chewing preferences, are necessary to monitor progressive structural changes, correlate clinical findings with bone imaging, and clarify the causal link between subtle TMJ alterations and OA. An additional limitation of the present study is that the morphometric measurements obtained by CBCT were restricted to osseous structures, without direct evaluation of the articular disc. Disc displacement and other discal alterations are known to influence joint biomechanics, modifying the direction and magnitude of loading forces on the condyle and glenoid fossa. Such changes may contribute to remodeling processes and could explain some of the morphometric variations observed in osteoarthritic joints. Since CBCT cannot assess disc position or integrity, our findings should be interpreted as structural correlates independent of discal status. Future investigations integrating CBCT with MRI would be particularly valuable to clarify the relationship between disc condition and morphometric parameters, thereby strengthening the clinical relevance of imaging-based predictors of TMJ osteoarthritis.

## Conclusions

CBCT imaging provides a reliable framework for identifying both established and early osteoarthritic changes in the TMJ of asymptomatic adults. Indeterminate cases, often marked by flattening or sclerosis, should be regarded as transitional stages that warrant closer monitoring.

## Data Availability

Data supporting the statements of the current article will be available to the editorial board upon request.
